# Gutta Percha Canal Points

**Published:** 1906-12

**Authors:** Gustavus North

**Affiliations:** Cedar Rapids, Iowa.


### GUTTA PERCHA CANAL POINTS.

By Dr. Gustavus North, Cedar Rapids, Iowa.

Gutta-percha points are generally too soft for practical use, except in favorable cases, as they often fail to follow the root canal and make an imperfect filling.

Making gutta-percha points is very simple, as follows: Cut the sheet gutta-percha into little pellets, then roll them on glass or marble slab with a warm steel spatula to any size desired, then dip each point in to sandarac or shellac varnish (the latter preferable) and lay them on glass, rolling them occasionally with the finger until dry to keep them from adhering to the glass.

The points, when dry, will be stiff and firm, and will follow any root canal, and by the use of chloro-percha will make a good filling.

